# An elusive diagnosis: IPSID presenting as a tuberculosis-like gastrointestinal disorder

**DOI:** 10.12669/pjms.42.(ICON26).15705

**Published:** 2026-04

**Authors:** Muzeer Ahmed, Abdul Nafey Kazi, Fivzia Farooq Herekar, Muhammad Junaid Patel

**Affiliations:** 1 Muzeer Ahmed, Department of Internal Medicine, Indus Hospital and Health Network, Karachi, Pakistan; 2 Abdul Nafey Kazi, Department of Internal Medicine, Indus Hospital and Health Network, Karachi, Pakistan; 3 Fivzia Farooq Herekar, Department of Internal Medicine, Indus Hospital and Health Network, Karachi, Pakistan; 4 Muhammad Junaid Patel, Department of Internal Medicine, Indus Hospital and Health Network, Karachi, Pakistan

**Keywords:** Abdominal tuberculosis, B-cell lymphoma, Immunoproliferative Small Intestinal Disease, Malabsorption

## Abstract

Immunoproliferative Small Intestinal Disease (IPSID) is a rare lymphoproliferative disorder involving the proximal small intestine with no definitive pathogenesis reported. It typically presents with chronic diarrhea, weight loss, and malabsorption, closely mimicking conditions such as abdominal tuberculosis (TB) and celiac disease. Reports from Pakistan are extremely uncommon. We report a 17 years old male with chronic diarrhea and weight loss, initially treated as abdominal TB for six months. Despite partial improvement, his symptoms relapsed with worsening weakness. Lymph node biopsy confirmed IPSID with transformation into B-cell Non-Hodgkin Lymphoma.

## INTRODUCTION

Immunoproliferative Small Intestinal Disease (IPSID) is a rare spectrum of lymphoproliferative diseases involving the small intestine (duodenum and jejunum) that may present in various clinical stages: benign, intermediate, or malignant.^1^ Initially discovered in the early 1960s, it was primarily considered to affect people living in the Mediterranean region.^2^ Low socio-economic status, poor sanitation, and unhealthy drinking water are among the risk factors associated with IPSID, especially in developing countries.^3^ Most patients with IPSID are young adults having ages 15-35 years with peak incidence in their second and third decades of life. An estimated ratio of male-to-female of 2:1 has been described in some reports.^4^ IPSID is quite rare with only four cases reported each year globally.

Pakistan is endemic for tuberculosis (TB) having an estimated incidence of 264 per 100,000 inhabitants in 2022, with an estimated 6-7% of cases consisting of abdominal TB that may or usually or mostly present with chronic diarrhea and weight loss.^5^ Both abdominal TB and IPSID have similar clinical presentations, but due to extreme rarity in Pakistan, abdominal TB is considered the primary diagnosis in such cases. We present a case of 17 years old South Asian male with a history of chronic diarrhea who was initially managed as abdominal TB but was later diagnosed with IPSID. This case report highlights the subtle, often nonspecific presentation of IPSID and demonstrates how delays in suspicion can postpone definitive treatment. It reinforces the importance of targeted workup particularly duodenal biopsy with immunophenotyping and appropriate imaging to avoid misdiagnosis.

## CASE PRESENTATION

A 17 years old male, with a poor-socio-economic status presented in the Emergency Department (ED) with complaints of worsening generalized weakness and loose stools for six weeks. He had no other significant past medical or surgical history. For the past 8-9 months, the patient complained of loose watery stools, yellow in color, not containing blood or mucus, 7-8 episodes per day with significant unintentional, undocumented weight loss, for which he was diagnosed with abdominal TB based on ultrasound findings of necrotic lymph nodes and started on first-line anti-tuberculous therapy (ATT). In the initial few months of ATT, he gained weight, and his diarrhea improved. But a few months later, he again complained of loose stools and weight loss despite being on ATT.

On initial examination in the ED, he was a young, thin-built male, lethargic looking with apparent temporal wasting and having blood pressure of 112/64mmHg, heart rate of 122/min with low volume pulse, respiratory rate of 20/minutes, oxygen saturation 98% at room air, and afebrile. He had pallor, clubbing, and multiple sub-centimeter lymph nodes in cervical, submandibular, and inguinal areas. Abdomen was soft, non-tender, distended with no visceromegaly or shifting dullness. The rest of the examination was unremarkable. He had a weight of 26kg. All initial blood workups were sent, and he was admitted for intravenous hydration and further workup.

During his hospital stay, he had anemia with a Hemoglobin level of 4.7mg/dl and was transfused two points of PCV. He had loose watery stools during admission, which were managed with intravenous hydration and electrolyte replacement. An initial impression of multi-drug resistance TB vs Celiac disease was made, and an extensive workup was done to exclude other causes including HIV and Hyperthyroidism. His TSH was normal and HIV serology was negative. His stool analysis was negative for ova and parasites, and celiac serology was negative. His serum albumin was low and he had trace pedal edema. Computed Tomography (CT) of the Abdomen was done, which reported significant mesenteric lymphadenopathy with a conglomerate mesenteric lymph nodal mass measuring 15.5 x 7.1 cm on coronal sections, and an increased number of ileal intestine folds were noted (Table-I).

**Table-I:** Laboratory workup for our patient.

Laboratory Tests		Laboratory tests	
Hb (13-16mg/dl)	4.7	TSH (0.5-4.5)	2.7
WBC (4-11 x10^9^ d/l)	10.6	ANA	Negative
Platelets (x10^9^ d/l)	416	ENA Profile	Negative
Serum Albumin (3.5-4g/dl)	2	HIV	Negative
ESR (mm/hr)	104	Celiac Serology	Negative
Stool Detailed Report	Negative for Ova and Parasites	LDH	304IU/L

Gastroenterology Department was consulted, and they did endoscopy and colonoscopy, which revealed duodenal and colonic ulcers with multiple biopsies sent. General surgery was consulted, and they did a laparoscopic lymph nodal biopsy of the mesenteric lymph nodal mass. All TB workups were reported negative (Table-II). He was discharged on oral rehydration salts and a soft diet with biopsies to be chased at the Outpatient Department (OPD) in two weeks.

**Table-II:** TB workup for our Patient.

AFB SMEAR GENE EXPERT	Sputum: Negative	Endoscopy biopsy: Negative	Lymph node Biopsy: Negative

In OPD, his duodenal biopsy revealed fragments of small intestinal mucosa exhibiting partial villous atrophy with surface epithelial damage by a monotonous lymphoplasmacytic infiltrate. His colonoscopy biopsy revealed fragments of small intestinal mucosa exhibiting total villous atrophy with surface epithelial damage by a monotonous lymphoplasmacytic infiltrate. His lymph nodal biopsy reported morphological and immunohistochemical features favoring B-Cell Non-Hodgkin Lymphoma or IPSID (Stage-C).

The family and the patient were counselled, and he was referred to the Oncology setup for further management. However, due to disease progression, he passed away a few weeks after his diagnosis.

## DISCUSSION

IPSID is a rare form of extranodal lymphoma, reported primarily in North Africa, the Middle East, and parts of Asia, but very uncommon in Pakistan. IPSID presents with typical features of malabsorption syndrome, including abdominal pain, chronic diarrhea, and weight loss. Local published data also reports similar symptom constellation dominated by diarrhea and abdominal pain.^6^ These nonspecific clinical features often mimic conditions such as abdominal tuberculosis and celiac disease, which can delay diagnosis, as occurred in our patient.^7^ Our patient was a young male from a low socioeconomic background presenting with diarrhea. Pervaiz et al and Malik et al described alike risk factors among IPSID patients in Pakistan that were young, predominantly male adults from lower socioeconomic backgrounds presenting with diarrhoea, malabsorption, and weight loss.^4^,^6^

Detailed literature reports no definitive pathogenesis of IPSID, but it has primarily been reported to occur due to chronic antigenic stimulation by recurrent or chronic gastrointestinal infections. Saleem et al reported an infectious etiology as the most common cause of IPSID.^8^ Despite this, no single causative agent has been conclusively identified, with various infectious agents reported, including enteric infections, giardiasis, and other parasitic infections, such as Trichuris trichiura.^9^

IPSID diagnosis requires a high index of suspicion in patients with chronic diarrhea and unexplained malabsorption. Endoscopy may reveal mucosal edema, erythema, nodularity, and ulceration, while histopathology typically demonstrates villous atrophy with a dense lymphoplasmacytic infiltrate.^7^ Our patient’s endoscopy revealed typical similar findings of multiple ulcers, with duodenal biopsy revealing partial villous atrophy with surface epithelial damage by a monotonous lymphoplasmacytic infiltrate. In Pervaiz et al.’s case series, IPSID diagnosis was done by upper endoscopy and small bowel biopsies with findings of dense plasmacytic infiltrate, villous blunting / atrophy and Lymphoepithelial lesions.^4^

IPSID in advanced stages can involve mesenteric and retroperitoneal lymph nodes, but solid organ infiltration is uncommon.^10^ In these cases, the disease may transform into diffuse large B-cell lymphoma (DLBCL), which has been associated with complications such as bowel obstruction and gastrointestinal hemorrhage.^11^ Our patient had advanced disease with transformation into B-cell lymphoma.

The clinical course varies depending on disease stage. The Galian staging system classifies IPSID into three stages (A, B, and C), based on the nature of infiltrate and extent of disease. Our patient had advanced disease and died before his treatment could be initiated in the Oncology setup. Early-stage disease often responds to prolonged oral antibiotics, sometimes leading to a cure. However, advanced disease typically requires systemic chemotherapy with cyclophosphamide, vincristine, doxorubicin, and prednisone (CHOP)-based regimens, often combined with antibiotics, achieving disease-free survival rates of 60–70% at three years.^8^

## CONCLUSION

Our case highlights the diagnostic challenge of IPSID in non-endemic regions and emphasizes the importance of early recognition. The occurrence of IPSID in Pakistan is likely under-recognized rather than truly rare, given the country’s high prevalence of chronic gastrointestinal infections, malnutrition, and low socioeconomic conditions factors known to predispose to MALT-related lymphomas in many endemic regions. A chronic history of diarrhea, prior abdominal tuberculosis, recurrent enteric infections, and long-standing nutritional deficiencies should therefore prompt clinicians to consider IPSID early in the differential diagnosis, rather than attributing such symptoms solely to infectious or inflammatory etiologies.

### Author contributions:

**MA and ANK:** Prepared the manuscript and are responsible for integrity of the study.

**FFH and MJP:** Literature search, critical reiew.

**ANK:** Responsible for the accuracy or integrity of the study.
